# Author Correction: scINSIGHT for interpreting single-cell gene expression from biologically heterogeneous data

**DOI:** 10.1186/s13059-022-02672-4

**Published:** 2022-04-21

**Authors:** Kun Qian, Shiwei Fu, Hongwei Li, Wei Vivian Li

**Affiliations:** 1 School of Mathematics and Physics, China University of Geosciences, Wuhan, 430074 Hubei China; 2 Department of Biostatistics and Epidemiology, Rutgers School of Public Health, Rutgers, The State University of New Jersey, Piscataway, NJ 08854 USA

**Correction to: Genome Biol 23, 82 (2022)**

**https://doi.org/10.1186/s13059-022-02649-3**

Following publication of the original article [1], the authors identified a missing reference and citation in the last paragraph of the section “The scINSIGHT model”. The last two sentences of this paragraph should read:

These limitations are resolved by scINSIGHT to better jointly analyze single-cell samples from different biological conditions. In addition, scINSIGHT is different from the CSMF method [2], which learns common and specific patterns among $J$ datasets from $J$ conditions.

The original article [1] has been corrected.
