# Supplementary material for: scINSIGHT for interpreting single-cell gene expression from biologically heterogeneous data
Source: Genome Biol. 2022 Mar 21;23:82. doi: 10.1186/s13059-022-02649-3 (PMC8935111; doi:10.1186/s13059-022-02649-3)
Supplement: Supplementary file 2 — Additional file 2 Supplementary methods. [file 13059_2022_2649_MOESM2_ESM.pdf]

Supplementary Methods

S1. Algorithm for solving the scINSIGHT model

The complete procedure for solving the scINSIGHT model is given in Algorithm 1. It is derived with the following major steps.

In the scINSIGHT model, we minimize the objective function $\mathcal{L}$ by iteratively solving

$$V(k,\cdot) \leftarrow \arg\min_{v} \ \mathcal{L}\left(\{W_{\ell 1}, W_{\ell 2}\}_{\ell=1}^{L}, \{H_j\}_{j=1}^{J}, V(1,\cdot), \ldots, V(k-1,\cdot), v, V(k+1,\cdot), \ldots, V(K,\cdot)\right) \ , \tag{s1}$$

for $k = 1, 2, \ldots, K$;

$$W_{\ell 2}(\cdot,k) \leftarrow \arg\min_{w} \ \mathcal{L}\left(\{W_{\ell 1}\}_{\ell=1}^{L}, \{W_{\ell' 2}\}_{\ell' \neq \ell}^{L}, \{H_j\}_{j=1}^{J}, V, W_{\ell 2}(\cdot,1), \ldots, W_{\ell 2}(\cdot,k-1), w, W_{\ell 2}(\cdot,k+1), \ldots, W_{\ell 2}(\cdot,K)\right) \ , \tag{s2}$$

for $k = 1, 2, \ldots, K, \ell = 1, 2, \ldots, L$;

$$W_{\ell 1}(\cdot,k) \leftarrow \arg\min_{w} \mathcal{L}\left(\{W_{\ell' 1}\}_{\ell'=1,\ell' \neq \ell}^{L}, \{W_{\ell 2}\}_{\ell=1}^{L}, \{H_j\}_{j=1}^{J}, V, W_{\ell 1}(\cdot,1), \ldots, W_{\ell 1}(\cdot,k-1), w, W_{\ell 1}(\cdot,k+1), \ldots, W_{\ell 1}(\cdot,k)\right) \tag{s3}$$

for $k = 1, 2, \ldots, K, \ell = 1, 2, \ldots, L$.

$$H_j(k,\cdot) \leftarrow \arg\min_{h} \mathcal{L}\left(\{W_{\ell 1}, W_{\ell 2}\}_{\ell=1}^{L}, V, \{H_j'\}_{j'=1,j' \neq j}^{J}, H_j(1,\cdot), \ldots, H_j(k-1,\cdot), h, H_j(k+1,\cdot), \ldots, H_j(K_j,\cdot)\right) \tag{s4}$$

for $k = 1, 2, \ldots, K_j, j = 1, 2, \ldots, J$.

The minimization problem of (s1)

The minimization problem of $V(k,\cdot)$ can be formulated as

$$\arg\min_{v \geq 0} \sum_{\ell=1}^{L} \frac{1}{m_\ell} \left\| W_{\ell 2}(\cdot,k) v^T - R_{\ell k}^{(V)} \right\|_F^2 ,$$

where

$$R_{\ell k}^{(V)} = X_\ell - W_{\ell 1} H_{j_\ell} - \sum_{k'=1,k' \neq k}^{K} W_{\ell 2}(\cdot,k') V(k',\cdot). \tag{s5}$$

Algorithm 1: The BCD algorithm for the scINSIGHT model

Input : $X_\ell \in \mathbb{R}_+^{m_\ell \times n}, K, K_j, \lambda_1 and \lambda_2$.

Output: $V \in \mathbb{R}_+^{K \times n}, W_{\ell 1} \in \mathbb{R}_+^{m_\ell \times K_{j_\ell}}, W_{\ell 2} \in \mathbb{R}_+^{m_\ell \times K}, H_j \in \mathbb{R}_+^{K_j \times n}$.

```
Initialize V, W_ℓ1, W_ℓ2 and H_j with random nonnegative values.
while not converged do
    for k = 1, ..., K do
        V(k,·) ← [Σ_{ℓ=1}^L (1/m_ℓ) (R_{ℓk}^{(V)})^T W_ℓ2(·,k)]_+ / (Σ_{ℓ=1}^L (1/m_ℓ) ||W_ℓ2(·,k)||_2^2).
    end
    for ℓ = 1, ..., L do
        for k = 1, ..., K do
            W_ℓ2(·,k) ← [W_ℓ2(·,k) + (X_ℓ − W_ℓ1 H_{j_ℓ} − W_ℓ2 V) V^T / ||V(k,·)||_2^2]_+.
            W_ℓ1(·,k) ← [W_ℓ1(·,k) + (X_ℓ − (1+λ1) W_ℓ1 H_{j_ℓ} − W_ℓ2 V) H_{j_ℓ}(k,·) / ((1+λ) ||H_{j_ℓ}(k,·)||_2^2)]_+.
        end
    end
    for j = 1, ..., J do
        for k = 1, ..., K_j do
            H_j(k,·) ←
              [H_j(k,·) + (Σ_{j_ℓ=j} (1/m_ℓ) (X_ℓ − (1+λ1) W_ℓ1 H_j − W_ℓ2 V)^T W_ℓ1(·,k) − (λ2/4) Σ_{j'≠j}^J Σ_{k'=1}^{K_{j'}} H_{j'}(k',·)) / (Σ_{j_ℓ=j} ((1+λ1)/m_ℓ) ||W_ℓ1(·,k)||_2^2)]_+.
            α = ||H_j(k,·)||_2.
            H_j(k,·) ← H_j(k,·) / α.
            // Adjust all W_ℓ1(·,k) satisfying j_ℓ = j.
            W_ℓ1(·,k) ← α W_ℓ1(·,k).
        end
    end
end
for k = 1, ..., K do
    β = ||V(k,·)||_2.
    V(k,·) ← V(k,·) / β.
    // Adjust all W_ℓ2(·,k).
    W_ℓ2(·,k) ← β W_ℓ2(·,k).
end
```

Line 4: $$V(k,\cdot) \leftarrow \frac{\left[\sum_{\ell=1}^{L} \frac{1}{m_\ell} \left(R_{\ell k}^{(V)}\right)^T W_{\ell 2}(\cdot,k)\right]_+}{\sum_{\ell=1}^{L} \frac{1}{m_\ell} \|W_{\ell 2}(\cdot,k)\|_2^2}.$$

Line 8: $$W_{\ell 2}(\cdot,k) \leftarrow \left[W_{\ell 2}(\cdot,k) + \frac{\left(X_\ell - W_{\ell 1} H_{j_\ell} - W_{\ell 2} V\right) V^T}{\|V(k,\cdot)\|_2^2}\right]_+.$$

Line 9: $$W_{\ell 1}(\cdot,k) \leftarrow \left[W_{\ell 1}(\cdot,k) + \frac{\left(X_\ell - (1+\lambda_1) W_{\ell 1} H_{j_\ell} - W_{\ell 2} V\right) H_{j_\ell}(k,\cdot)}{(1+\lambda)\left\|H_{j_\ell}(k,\cdot)\right\|_2^2}\right]_+.$$

Line 14: $$H_j(k,\cdot) \leftarrow \left[H_j(k,\cdot) + \frac{\sum_{j_\ell=j} \frac{1}{m_\ell} (X_\ell - (1+\lambda_1) W_{\ell 1} H_j - W_{\ell 2} V)^T W_{\ell 1}(\cdot,k) - \frac{\lambda_2}{4} \sum_{j' \neq j}^{J} \sum_{k'=1}^{K_{j'}} H_{j'}(k',\cdot)}{\sum_{j_\ell=j} \frac{1+\lambda_1}{m_\ell} \|W_{\ell 1}(\cdot,k)\|_2^2}\right]_+.$$

Consider a minimization problem

$$\min_{v\geq 0}\sum_{\ell=1}^{L}\frac{1}{m_\ell}\left\|u_\ell v^T-G_\ell\right\|_F^2,$$

where $G_\ell\in\mathbb{R}^{M_\ell\times N}$ and $u_\ell\in\mathbb{R}^{M_\ell\times 1}$ ($\ell=1,2,\ldots,L$). We further denote $v^T=(v_1,v_2,\ldots,v_N)$ and $G_\ell=[g_{\ell 1},g_{\ell 2},\ldots,g_{\ell N}]$. Since $v\geq 0$, we have

$$\min_{v\geq 0}\sum_{\ell=1}^{L}\frac{1}{m_\ell}\left\|u_\ell v^T-G_\ell\right\|_F^2=\sum_{n=1}^{N}\min_{v_n\geq 0}\sum_{\ell=1}^{L}\frac{1}{m_\ell}\left\|u_\ell v_n-g_{\ell n}\right\|_2^2,$$

where the minimum is achieved at

$$v_n=\max\left\{0,\frac{\sum_{\ell=1}^{L}\frac{1}{m_\ell}u_\ell^T g_{\ell n}}{\sum_{\ell=1}^{L}\frac{1}{m_\ell}\|u_\ell\|_2^2}\right\}=\frac{\left[\sum_{\ell=1}^{L}\frac{1}{m_\ell}u_\ell^T g_{\ell n}\right]_+}{\sum_{\ell=1}^{L}\frac{1}{m_\ell}\|u_\ell\|_2^2}.$$

Therefore, the solution of (s1) is

$$V(k,\cdot)\leftarrow\frac{\left[\sum_{\ell=1}^{L}\frac{1}{m_\ell}\left(R_{\ell k}^{(V)}\right)^T W_{\ell 2}(\cdot,k)\right]_+}{\sum_{\ell=1}^{L}\frac{1}{m_\ell}\|W_{\ell 2}(\cdot,k)\|_2^2}. \tag{s6}$$

Replacing $R_{\ell k}^{(V)}$ in (s6) with expression in (s5), we have

$$V(k,\cdot)\leftarrow\left[V(k,\cdot)+\frac{\sum_{\ell=1}^{L}\frac{1}{m_\ell}\left(X_\ell-W_{\ell 1}H_{j_\ell}-W_{\ell 2}V\right)^T W_{\ell 2}(\cdot,k)}{\sum_{\ell=1}^{L}\frac{1}{m_\ell}\|W_{\ell 2}(\cdot,k)\|_2^2}\right]_+.$$

The minimization problem and solution of (s2)

The minimization problem of $W_{\ell 2}(\cdot,k)$ appears as

$$\min_{w\geq 0}\frac{1}{m_\ell}\left\|V(k,\cdot)w^T-\left(R_{\ell k}^{V}\right)^T\right\|_F^2.$$

Using a similar approach as above, the solution of (s2) is

$$W_{\ell 2}(\cdot,k)\leftarrow\frac{\left[R_{\ell k}^{(V)}V(k,\cdot)\right]_+}{\|V(k,\cdot)\|_2^2}. \tag{s7}$$

Replacing $R_{\ell k}^{(V)}$ in (s7) with expression in (s5), we have

$$W_{\ell 2}(\cdot,k)\leftarrow\left[W_{\ell 2}(\cdot,k)+\frac{\left(X_\ell-W_{\ell 1}H_{j_\ell}-W_{\ell 2}V\right)V^T}{\|V(k,\cdot)\|_2^2}\right]_+.$$

The minimization problem and solution of (s3)

The minimization problem of $W_{\ell 1}(k, \cdot)$ appears as

$$\min_{w\geq 0} \frac{1}{m_\ell} \left\| H_{j_\ell}(k,\cdot) w^T - \left(R_{\ell k}^{(H)}\right)^T \right\|_F^2 + \frac{\lambda}{m_\ell} \left\| H_{j_\ell}(k,\cdot) w^T + S_{\ell k}^T \right\|_F^2$$

where

$$S_{\ell k} = \sum_{k'=1, k'\neq k}^{K_{j_\ell}} W_{\ell 1}(\cdot, k') H_{j_\ell}(k', \cdot)^T, \tag{s8}$$

$$R_{\ell k}^{(H)} = X_\ell - W_{\ell 2} V - S_{\ell k}. \tag{s9}$$

Using a similar approach as above, the solution of (s3) is

$$W_{\ell 1}(\cdot, k) \leftarrow \frac{\left[\left(R_{\ell k}^{(H)} - \lambda S_{\ell k}\right) H_{j_\ell}(k,\cdot)\right]_+}{(1+\lambda) \left\| H_{j_\ell}(k,\cdot)\right\|_2^2}\ . \tag{s10}$$

Replacing $S_{\ell k}$ and $R_{\ell k}^{(H)}$ in (s10) with expression in (s8) and (s9), the solution can be rewritten as

$$W_{\ell 1}(\cdot, k) \leftarrow \left[W_{\ell 1}(\cdot, k) + \frac{\left(X_\ell - (1+\lambda) W_{\ell 1} H_{j_\ell} - W_{\ell 2} V\right) H_{j_\ell}(k,\cdot)}{(1+\lambda)\left\| H_{j_\ell}(k,\cdot)\right\|_2^2} \right]_+ .$$

The minimization problem and solution of (s4)

The minimization problem of $H_j(k,\cdot)$ appears as

$$\min_{h\geq 0} \sum_{j_\ell = j} \frac{1}{m_\ell} \left[\left\| W_{\ell 1}(\cdot, k) h^T - R_{\ell k}^{(H)} \right\| + \lambda_1 \left\| W_{\ell 1}(\cdot,k) h^T + S_{\ell k}\right\|_F^2 \right] + \frac{\lambda_2}{2} h^T \sum_{j'=1, j'\neq j}^{J} \sum_{k'=1}^{K_{j'}} H_{j'}(k', \cdot).$$

Consider a minimization problem

$$\min_{v\geq 0} \sum_{i\in I} \frac{1}{m_i} \left[\left\| u_i v^T - G_i \right\|_F^2 + \lambda_1 \left\| u_i v^T + Q_i \right\|_F^2 \right] + \lambda_2 v^T w,$$

where $G_i, Q_i \in \mathbb{R}^{M_i \times N}, u_i \in \mathbb{R}^{m_i \times 1}, i \in I$ and $H_j \in \mathbb{R}^{K_j \times N}$ are given. Let $v^T = (v_1, 2, \ldots, v_N), G_i = [g_{i,1}, \ldots, g_{i,N}], Q_i = [q_{i,1}, \ldots, q_{i,N}]$ and $H_j = [h_{j,1}, \ldots, h_{j,N}]$. Since $v \geq 0$, we have

$$\begin{aligned} &\min_{v\geq 0} \sum_{i\in I} \frac{1}{m_i} \left[\left\| u_i v^T - G_i \right\|_F^2 + \lambda_1 \left\| u_i v^T + Q_i \right\|_F^2 \right] + \lambda_2 v^T w \\ &= \sum_{n=1}^{N} \min_{v_n \geq 0} \sum_{i\in I} \frac{1}{m_i} \left[\left\| u_i v_n - g_{i,n} \right\|_2^2 + \lambda_1 \left\| u_i v_n + q_{i,n}\right\|_2^2 \right] + \lambda_2 v_n w_n. \end{aligned}$$

Solving the above problem, that the solution of (s4) can be obtained:

$$H_j(k,\cdot) \leftarrow \left[H_j(k,\cdot) + \frac{\sum_{j_\ell=j} \frac{1}{m_\ell} \left(R_{\ell k}^{(H)} - \lambda_1 S_{\ell k} \right)^T W_{\ell 1}(\cdot,k) - \frac{\lambda_2}{4} \sum_{j'=1,j'\neq j}^{J} \sum_{k'=1}^{K_{j'}} H_{j'}(k',\cdot)}{\sum_{j_\ell=j} \frac{1+\lambda_1}{m_\ell} \|W_{\ell 1}(\cdot,k)\|_2^2} \right]_+ . \tag{s11}$$

Replacing $S_{\ell k}$ and $R_{\ell k}^{(H)}$ in (s11) with expression in (s8) and (s9) we have

$$H_j(k,\cdot) \leftarrow \left[H_j(k,\cdot) + \frac{\sum_{j_\ell=j} \frac{1}{m_\ell} \left(X_\ell - (1+\lambda_1) W_{\ell 1} H_j - W_{\ell 2} V \right)^T W_{\ell 1}(\cdot,k) - \frac{\lambda_2}{4} \sum_{j'=1,j'\neq j}^{J} \sum_{k'=1}^{K_{j'}} H_{j'}(k',\cdot)}{\sum_{j_\ell=j} \frac{1+\lambda_1}{m_\ell} \|W_{\ell 1}(\cdot,k)\|_2^2} \right]_+ .$$

After we update $H_j(k,\cdot)$, we need to calculate the $L2$ norm of $H_j(k,\cdot)$ as $\alpha = \|H_j(k,\cdot)\|_2$, then normalize it by

$$H_j(k,\cdot) \leftarrow \frac{H_j(k,\cdot)}{\alpha} ,$$

and adjust all $W_{\ell 1}(\cdot,k)$ satisfying $j_\ell = j$ by

$$W_{j_\ell}(\cdot,k) \leftarrow \alpha W_{j_\ell}(\cdot,k) .$$

After we finish the iteration, we need to calculate the $L2$ norm of $V(k,\cdot)$ as $\beta = \|V(k,\cdot)\|_2$, then normalize it by

$$V(k,\cdot) \leftarrow \frac{V(k,\cdot)}{\beta} ,$$

and adjust all $W_{\ell 2}(\cdot,k)$ by

$$W_{\ell 2}(\cdot,k) \leftarrow \beta W_{\ell 2}(\cdot,k) ,$$

for $\ell = 1,2,\ldots,L, k = 1,2,\ldots,K$.
