# Supplementary material for: scINSIGHT for interpreting single-cell gene expression from biologically heterogeneous data
Source: Genome Biol. 2022 Mar 21;23:82. doi: 10.1186/s13059-022-02649-3 (PMC8935111; doi:10.1186/s13059-022-02649-3)
Supplement: Supplementary file 3 — Additional file 3 Review history. [file 13059_2022_2649_MOESM3_ESM.docx]

Review History

**First round of review**

**Reviewer 1**

**Were you able to assess all statistics in the manuscript, including the appropriateness of statistical tests used?**

Yes

**Comments to Authors:**

scINSIGHT for interpreting single-cell gene expression from biologically heterogeneous data

In this manuscript, Qian et al. describe the development of a non-negative matrix factorization technique which accounts for the presence of "common" and "condition-specific" gene pathways, important for when biological variation across datasets is confounded with technical variation. They then use this framework to evaluate simulated data, as well as a variety of real-world data, including melanoma, COVID-19, and wound healing. scINSIGHT appears to function well for batch correction and joint analysis of heterogeneous samples, thus fulfilling the primary aim described by the authors. Overall, the manuscript is well-structured and well-written, but would benefit from some changes as outlined below -

Major points:

1. The manuscript does not provide an intuitive explanation of how the algorithm proposed classifies a specific gene pathway as being "common" or "condition-specific". How are these distinctions made, and what parameters/thresholds are being used for this classification of pathways into one or the other category?

2. The specific use-case for the tool described in this manuscript is not explicitly stated. While the authors mention that it is meant for batch correction and joint clustering in cases where samples derive from "distinct but relevant" biological conditions, this characterization would benefit from further formalization. As the authors undoubtedly are aware, there is a glut of batch correction and integration frameworks currently available for single-cell data, and a clear statement about specific use-cases would also help to further distinguish this tool from pre-existing software using NMF such as LIGER.

3. Since highly variable genes (HVGs) often perform as well as known marker, it would be interesting to investigate the use of HVGs in the melanoma dataset (as opposed to the CD8+ gene signatures which are used in the manuscript) as was done for the COVID-19 and wound healing datasets for initial feature selection.

4. Some greater evaluation of the overall co-expression of the derived gene sets would be useful. There are a variety of methods that could be applied to provide some (post-hoc) sense of the biological validity of the results.

5. More empirical modeling might be helpful. PMID=29491377 takes an approach that might be interesting here, of sampling genes and swapping them into otherwise distinct cellular profiles (at various thresholds for specificity).

Other/miscellaneous:

1. The software as described in the vignette does not run as expected - the run_scINSIGHT function returns an error ("Error in x[["W_2"]] : subscript out of bounds"), for the simulated as well as the real data, rendering it impossible to actually use the tool on other real-world data.

2. In Figure 5, the "condition-specific" genes in the heatmap in (G) are not genes typically associated with B-cell responses (e.g. GNLY/NKG7), and includes highly/constitutively-expressed genes such as MALAT1 and GAPDH. Furthermore, the scale for the heatmap is mirrored.

**Reviewer 2**

**Were you able to assess all statistics in the manuscript, including the appropriateness of statistical tests used?**

Yes

**Comments to author:**

In this work, the authors aimed to analyze "heterogeneous single-cell samples coming from distinct but relevant biological conditions", Towards this they proposed a method called, scINSIGHT, a non-negative matrix factorization approach that identifies both coordinated gene expression patterns that are common among and that specific to different biological conditions. The reviewers have the following comments:

1. The mathematical frame work of scINSIGHT is not novel, it is very similar to that of LIGER, if not identical. Can the authors further explain the uniqueness of the mathematical framework?

2. This manuscript focuses on identifying common and specific gene pathways (rather than genes like other methods), however, I was not able to find descriptions in the Methods on how exactly the genes are transformed into pathways. Also, since this method works at pathway level, it only seems to be fair when scINSIGHT is also compared to other methods where the genes are transformed to pathways as a pre-condition as well. I am not sure if this is the case when the comparisons were made on LIGER and Seurat.

3. Incomplete benchmarking: the authors stated that they chose LIGER and Seurat methods because of the recommendations from this earlier benchmark paper: Hoa Thi Nhu Tran, Kok Siong Ang, Marion Chevrier, Xiaomeng Zhang, Nicole Yee Shin Lee, Michelle Goh, and Jinmiao Chen. A benchmark of batch-effect correction methods for single cell rna sequencing data. Genome biology, 21(1):1-32, 2020. However, a closer look at this earlier paper's summary comparison Fig 21 shows that the methods seem to fall into 2 categories. The best methods include: Harmony, LIGER, Seurate 3, scGen, Scanorama, scMerge, from the order of the best to the (relatively) worst in the same class. The authors need to compare with these methods at least, and also on more comprehensive scenarios like this benchmark paper, using their datasets included in Scenario 2: non-identical cell types (dataset 1, dataset 7), scenario 3: multiple studies in dataset (dataset 4). This will put scINSIGHT in better perspectives for its performance. In addition, there are other NMF based methods, such as scCoGAPS (see paper: Decomposing Cell Identity for Transfer Learning across Cellular Measurements, Platforms, Tissues, and Species - PubMed (nih.gov)) and preprint (sc-JNMF): SC-JNMF: Single-cell clustering integrating multiple quantification methods based on joint non-negative matrix factorization | bioRxiv. A moe comprehensive comparison among different variants of NMF methods are also needed.

4. I do not see the comparison on the practical matters, such as running time and memory uses. I do not see how well this method will scale up for larger datasets.

5. Simulation data are simple cases, need more variations. In simulation data, what happens when the shared cell types is minimal? For example, if only 1 out of 5 cell types are shared? Also, what happens if you change the proportions of the different cell types and make some more common and some cell types rare?

**Authors Response**

**Point-by-point responses to the reviewers’ comments:**

Reviewer #1: In this manuscript, Qian et al. describe the development of a non-negative matrix factorization technique which accounts for the presence of "common" and "condition-specific" gene pathways, important for when biological variation across datasets is confounded with technical variation. They then use this framework to evaluate simulated data, as well as a variety of realworld data, including melanoma, COVID-19, and wound healing. scINSIGHT appears to function well for batch correction and joint analysis of heterogeneous samples, thus fulfilling the primary aim described by the authors. Overall, the manuscript is well-structured and well-written, but would benefit from some changes as outlined below.

*Response: We thank the reviewer for the positive comments and constructive suggestions on our work. We have revised the manuscript based on the reviewers’ suggestions, and the revised contents are highlighted in red in the updated manuscript. Please see our point-by-point response below.*

Major points: 1. The manuscript does not provide an intuitive explanation of how the algorithm proposed classifies a specific gene pathway as being "common" or "condition-specific". How are these distinctions made, and what parameters/thresholds are being used for this classification of pathways into one or the other category?

*Response: We apologize for this confusion. As introduced in the first paragraph of Results (page 3), scINSIGHT defines each gene pathway as a sparse and non-negative linear combination of genes, and each cell is jointly defined by the expression of common and condition-specific gene pathways. Since these pathways are automatically obtained from the matrix factorization results (model (2) in Methods), there is no need to manually classify them as being “common” or “condition-specific”. Given the reviewer’s question, we realize that the term of “pathway” could be confused with the existing biological pathways in public databases, so we have changed the term from “gene pathway” to “gene module” throughout the revised manuscript.*

2. The specific use-case for the tool described in this manuscript is not explicitly stated. While the authors mention that it is meant for batch correction and joint clustering in cases where samples derive from "distinct but relevant" biological conditions, this characterization would benefit from further formalization. As the authors undoubtedly are aware, there is a glut of batch correction and integration frameworks currently available for single-cell data, and a clear statement about specific use-cases would also help to further distinguish this tool from preexisting software using NMF such as LIGER.

*Response: We thank the reviewer’s suggestion to better distinguish scINSIGHT from existing methods and tools. Even though multiple batch correction and integration methods have been developed for scRNA-seq data, scINSIGHT is the first and only method which aims to jointly analyze multiple single-cell gene expression samples that can be grouped based on biological conditions. These biological conditions can be assigned based on sample phenotypes or experimental conditions, such as different developmental stages, disease phases, or treatment groups. While existing methods only use batch information in the batch correction or integration process, scINSIGHT can directly account for group information to analyze gene expression patterns that are common in or specific to different biological groups. We have added clarifications in both Introduction and Methods (pages 2, 3, and 17) of the revised manuscript. In the Discussion section, we have also explained that scINSIGHT aims at integration across biological groups, and is not intended to replace existing batch-effect removal tools. If it is known that the single-cell samples are from same biopsies or cell lines and the main difference lies in the sequencing platform or library preparation protocol, then it would suffice to use existing batch-effect removal methods to remove unwanted technical variation from the gene expression data.*

*In addition, to distinguish from the iNMF1 framework used in the LIGER method, we added an introduction to iNMF’s mathematical formulation on page 19 of the revised manuscript. Unlike the scINISGHT model, iNMF has the following limitations:*

*(1) It does not account for the assignment of biological conditions, so the model can only tell whether samples are from different batches, but cannot distinguish if they are from the same or different biological conditions;*

*(2) Unlike scINSIGHT that focuses on common and condition-specific gene modules, iNMF focuses on common and dataset-specific modules, and it requires that there exists the same number of common and dataset-specific modules;*

*(3) Dataset-specific gene modules by iNMF might capture both technical and biological signals, but condition-specific gene modules by scINSIGHT will only capture biological signals; These limitations are resolved by scINSIGHT to better jointly analyze single-cell samples from different biological conditions. We hope this newly added discussion has highlighted the unique advantages and contributions of scINSIGHT.*

3. Since highly variable genes (HVGs) often perform as well as known marker, it would be interesting to investigate the use of HVGs in the melanoma dataset (as opposed to the CD8+ gene signatures which are used in the manuscript) as was done for the COVID-19 and wound healing datasets for initial feature selection.

*Response: We thank the reviewer’s suggestion. We first added three additional methods in the comparison using CD8+ gene signatures, according to Reviewer 2’s suggestion. Then, we added an analysis to investigate the use of highly variable genes in the melanoma application, comparing scINSIGHT with the five alternative methods. Overall, the two sets of analysis showed similar results. Using highly variable genes, scINSIGHT still identified six clusters, although the separation between the clusters was slightly reduced compared with using CD8+ gene signatures (Figure R1). Using either highly variable genes or CD8+ gene signatures, scINSIGHT showed the best performance in integration and identification of cellular identities. The above new analyses were described on pages 7-9 of the revised manuscript.*

4. Some greater evaluation of the overall co-expression of the derived gene sets would be useful. There are a variety of methods that could be applied to provide some (post-hoc) sense of the biological validity of the results.

*Response: We thank the reviewer’s suggestion to investigate the co-expression of the inferred gene modules. Previously, we used gene ontology or pathway enrichment analysis to investigate the potential biological functions of the identified gene modules, and we agree that the coexpression analysis suggested by the reviewer would be complementary to our existing results. For each real dataset, we performed a co-expression analysis of the common and conditionspecific gene modules identified by scINSIGHT. For each gene module, we selected the top 25 genes with the largest coefficients, and used these genes to calculate within-module and between-module co-expression scores. The within-module co-expression in one condition was calculated as the average pairwise Spearman’s correlation of the top genes in that module, using all cells from that condition. The between-module co-expression in one condition was calculated as the average Spearman’s correlation between top genes in the two modules, using all cells from that condition.*

*Overall, we found that within-module co-expression was stronger than between-module coexpression, showing that gene modules identified by scINSIGHT tended to be co-expressed and functionally related. For example, the 13 common gene modules and 6 condition-specific gene modules identified from the COVID-19 dataset showed greater within-module coexpression than between-module co-expression (Figure R2). In a few cases, two common gene modules could also have a relatively high co-expression (e.g., common modules 3 and 9), indicating that they have coordinated roles in biological processes. The complete results are described on pages 10, 12, and 14 of the revised manuscript.*

5. More empirical modeling might be helpful. PMID=29491377 takes an approach that might be interesting here, of sampling genes and swapping them into otherwise distinct cellular profiles (at various thresholds for specificity)

*Response: We thank the reviewer for the suggestion to perform more empirical modeling to evaluate the robustness and accuracy of scINSIGHT. Since it is not clear how the gene sampling and swapping approach can be used in our studies, we followed Reviewer 2’s advice and added three sets of analysis based on different variants of the simulation study, which helped us evaluate scINSIGHT’s accuracy and robustness in different scenarios. In summary, we designed three variants of the original simulation data. Variant 1: only one cell type (C1) was shared by the single-cell samples; Variant 2: there existed a rare common cell type (the cell number of cell type C2 was reduced from 100 to 20 in each sample); Variant 3: there existed a rare condition-specific cell type (the cell number of cell type C4 was reduced from 100 to 20). Overall, scINSIGHT has demonstrated high accuracy and robustness in these additional simulation studies. Please see our response to Reviewer 2’ comment 5 for a detailed description.*

Other/miscellaneous: 1. The software as described in the vignette does not run as expected - the run_scINSIGHT function returns an error ("Error in x[["W_2"]] : subscript out of bounds"), for the simulated as well as the real data, rendering it impossible to actually use the tool on other real-world data.

*Response: We thank the reviewer for helping us test the scINSIGHT package, and we apologize for the errors. We have updated the package and tested it on both Linux and Mac OS systems.*

2. In Figure 5, the "condition-specific" genes in the heatmap in (G) are not genes typically associated with B-cell responses (e.g. GNLY/NKG7), and includes highly/constitutively-expressed genes such as MALAT1 and GAPDH. Furthermore, the scale for the heatmap is mirrored.

*Response: We thank the reviewer for the comment and suggestion on Figure 5. The heatmap in Figure 5G actually shows the relative expression of genes in common gene modules instead of conditionspecific gene modules. We have corrected the legend of the heatmap. We reviewed relevant literature and found that GNLY and NKG7 were used as the signature genes of natural killer cells in the original publication of the COVID-19 dataset. Given recent discovery of lymphocytes harboring phenotypic traits typical of both B cells and natural killer cells 2, there are two possible explanations of relatively high expression of GNLY/NKG7 in cluster C6: (1) cluster C6 represented a B cell population that also carried characteristics of natural killer cells; or (2) the original work annotated a natural killer population as B cells by mistake. We have added a discussion on page 12 to refine our interpretation. In addition, MALAT1 and GAPDH had relative high expression in cluster C3 (naïve B cell) and C10 (plasma B cell), respectively. This result does not suggest that they are associated with B-cell responses.*

Reviewer #2: In this work, the authors aimed to analyze "heterogeneous single-cell samples coming from distinct but relevant biological conditions", Towards this they proposed a method called, scINSIGHT, a non-negative matrix factorization approach that identifies both coordinated gene expression patterns that are common among and that specific to different biological conditions. The reviewers have the following comments:

*Response: We thank the reviewer for the summary of our work. We also appreciate the insightful comments and suggestions provided by the reviewer. We have revised the manuscript based on the reviewers’ suggestions, and the revised contents are highlighted in red in the updated manuscript. Please see our point-by-point response below.*

1. The mathematical frame work of scINSIGHT is not novel, it is very similar to that of LIGER, if not identical. Can the authors further explain the uniqueness of the mathematical framework?

*Response: We thank the reviewer’s suggestion to better explain the uniqueness of scINSIGHT compared with existing methods. Even though multiple batch correction and integration methods have been developed for scRNA-seq data, scINSIGHT is the first and only method which aims to jointly analyze multiple single-cell gene expression samples that can be grouped based on biological conditions. These biological conditions can be assigned based on sample phenotypes or experimental conditions, such as different developmental stages, disease phases, or treatment groups. While existing methods only use batch information in the batch correction or integration processes, scINSIGHT can directly account for group information to analyze gene expression patterns that are common in and specific to different biological groups. The condition-specific gene modules are modeled by the condition-specific H matrices in scINSIGHT’s factorization model (model (2) on page 18 of the revised manuscript). This formulation is uniquely proposed by scINSIGHT and not used by existing factorization methods. We have added clarifications in both Introduction and Methods (pages 2, 3, and 17) of the revised manuscript. The mathematical framework of scINSIGHT is different from the iNMF framework (used in the LIGER method) from multiple aspects. To distinguish from the iNMF framework, we added an introduction to iNMF’s mathematical formulation on page 19 of the revised manuscript. Unlike the scINSIGHT model, iNMF has the following limitations:*

*(1) It does not account for the assignment of biological conditions, so the model can only tell whether samples are from different batches, but cannot distinguish if they are from the same or different biological conditions;*

*(2) Unlike scINSIGHT that focuses on common and condition-specific gene modules, iNMF focuses on common and dataset-specific modules, and it requires that there exists the same number of common and dataset-specific modules;*

*(3) Dataset-specific gene modules by iNMF might capture both technical and biological signals, but condition-specific gene modules by scINSIGHT will only capture biological signals;*

*These limitations are resolved by scINSIGHT to better jointly analyze single-cell samples from different biological conditions. We hope this newly added discussion has highlighted the unique advantages and contributions of scINSIGHT.*

2. This manuscript focuses on identifying common and specific gene pathways (rather than genes like other methods), however, I was not able to find descriptions in the Methods on how exactly the genes are transformed into pathways. Also, since this method works at pathway level, it only seems to be fair when scINSIGHT is also compared to other methods where the genes are transformed to pathways as a pre-condition as well. I am not sure if this is the case when the comparisons were made on LIGER and Seurat.

*2. This manuscript focuses on identifying common and specific gene pathways (rather than genes like other methods), however, I was not able to find descriptions in the Methods on how exactly the genes are transformed into pathways. Also, since this method works at pathway level, it only seems to be fair when scINSIGHT is also compared to other methods where the genes are transformed to pathways as a pre-condition as well. I am not sure if this is the case when the comparisons were made on LIGER and Seurat.*

2. This manuscript focuses on identifying common and specific gene pathways (rather than genes like other methods), however, I was not able to find descriptions in the Methods on how exactly the genes are transformed into pathways. Also, since this method works at pathway level, it only seems to be fair when scINSIGHT is also compared to other methods where the genes are transformed to pathways as a pre-condition as well. I am not sure if this is the case when the comparisons were made on LIGER and Seurat.

*Response: We thank the reviewer for the above constructive suggestions on our benchmarking analysis. As the reviewer suggested, we have added benchmarking results based on additional methods and data in the revised manuscript. For the comparison with additional methods, we newly obtained integration results of five methods, Harmony, scMerge, scGen, Scanorama, and scCoGAPS. We tried to also include the SC-JNMF method, but it was only designed for joint analyses of two single-cell datasets and is not applicable to the problems discussed in our work. Although we were not able to compare scINSIGHT with SC-JNMF, the LIGER method, which we already compared with in the original work, is based on the popular iNMF method. The comparison with LIGER and scCoGAPS shows how scINSIGHT performs compared with the state-of-the-art NMF-based methods.*

*The new comparative analysis based on the simulated data shows that scINSIGHT and Scanorama achieved the highest adjusted Rand index and integration score among all the methods (see Figure R3 on the next page). In addition, we would also like to emphasize that scINSIGHT is the only method among the eight that is able to directly account for biological conditions and identify condition-specific gene modules.*

*In this analysis, we noticed that scCoGAPS only had an adjusted Rand index of 0.02 (Figure R3I). We studied the corresponding publication, Stein-O’Brien et al.3 , and found that unlike the other methods included in the comparison, scCoGAPS does not directly take multiple datasets as input. Instead, Stein-O’Brien et al. used a method named ProjectR to project new datasets to the latent space identified by scCoGAPS (using a source dataset). Since this approach requires the selection of a source dataset, which is not straightforward in applications studied in our work, we had to combine the datasets into a single count matrix before applying scCoGAPS. Given this difference between scCoGAPS and the other methods, we have decided to not include its results in the manuscript to avoid confusion. Since both scCoGAPS and SC-JNMF are relevant NMF-based methods, we have cited them in our revised manuscript (page 3), and we thank the reviewer for bring these methods to our attention. The revised simulation study is described on pages 5-7 of the revised manuscript.*

*In addition, we also compared scINSIGHT and the newly added methods based on the real datasets. For the melanoma dataset, scINSIGHT’s results showed a clearer separation of the clusters than the other methods (Figure R4 on next page). In addition, scINSIGHT achieved the highest integration and Silhouette scores (Figure R4). We also compared the computationally inferred cell clusters with the cell states annotated in the original publication, and found that scINSIGHT led to the best consistency (Figures S12-S13 in the Supplementary file). In terms of identifying cell clusters enriched in specific conditions, scINSIGHT also achieved the best sensitivity (Figure 4A in the revised manuscript). These new results are described on pages 7- 10 of the revised manuscript.*

*For the COVID-19 dataset, which has annotated cell type labels inferred by SingleR, we compared the methods based on the adjusted Rand index of inferred cell clusters. The integrated data by scINSIGHT again achieved the highest clustering accuracy (Figure R5). In the tSNE plots of integrated data, scINSIGHT also led to clearer separation of naïve B, memory B, and plasma B cells. It is worth noting that, since the clusters identified from single-cell data could correspond to B cell subtypes not available in the bulk reference, it was expected that the overall adjusted Rand index was not very high. These new results and the biological interpretations of condition-enriched clusters or condition-specific gene modules identified by scINSIGHT are described on pages 10-12 of the revised manuscript.*

*For the wound healing dataset, we also compared scINSIGHT with Seurat, LIGER, Harmony, scMerge, scGen, and Scanorama. scINSIGHT achieved the highest Silhouette scores among all the methods, and the second highest integration score after scMerge (Figure R6). Nevertheless, the low Silhouette scores of scMerge suggest that it removed biologically meaningful signals during integration. These new results are described on pages 14-15 of the revised manuscript.*

*Regarding the reviewer’s suggestion to evaluate scINSIGHT on Datasets 1, 4, and 7 from Tran et al., we would like to point out that Tran et al. aims at evaluating the methods’ performance on batch effect removal, so most datasets used in this paper are not all suitable for the application of scINSIGHT. Specifically, Dataset 1 contains cells from the same population but sequenced in different batches; Dataset 4 contains cells from the same healthy tissue type but sequenced using different protocols; Dataset 7 contains cells from the same healthy tissue type but sequenced by two independent laboratories. Therefore, in these three datasets, the differences in single-cell samples came from technical sources instead of biological sources, and the singlecell samples cannot be categorized by biological conditions for the application of scINSIGHT. We have clarified this point in the Discussion section:*

*“We would like to point out that scINSIGHT is not intended to replace existing batch-effect removal tools. If it is known that the single-cell samples are from the same tissue types, biopsies, 14 or cell lines, and the main difference lies in the sequencing platform or library preparation protocol, then it would suffice to use existing batch-effect removal or integration methods to remove unwanted technical variation from the gene expression data.”*

*We appreciate the reviewer’s suggestion to evaluate scINSIGHT in different scenarios. Our newly added simulation studies now reflect the scenarios with non-identical cell types or rare cell types (please see our response to Comment 5 for details). In addition, even though Dataset 7 from Tran et al. is not ideal for scINSIGHT’s application, we added a comparison based on this dataset to address the reviewer’s Comment 4 about scINSIGHT’s performance on largerscale data. Please see our response to Comment 4 for details.*

4. I do not see the comparison on the practical matters, such as running time and memory uses. I do not see how well this method will scale up for larger datasets.

*Response: We thank the reviewer for raising this important question and have added the information of running time and memory for all methods and datasets discussed in the revised manuscript. Our analysis was based on the CentOS 7.8.2003 system and Intel(R) Xeon(R) Gold 5218R processor (16 cores).*

*Overall, scINSIGHT’s memory usage is on the medium level among the seven methods (Table R1). scINSIGHT is admittedly slower than the other methods, and we summarize the major reasons below. First, it requires a more complex model to distinguish between technical signals, common biological signals, and condition-specific biological signals. Compared with LIGER which is also an NMF-based method, scINSIGHT’s optimization is naturally more complex and takes more time. Second, scINSIGHT includes internal steps to identify optimal parameters, so its running time is expectedly larger than methods using given parameters. For example, LIGER has a function to select the dimensionality of factorized matrices, but this function encountered errors for multiple datasets, and we had to directly input the parameters in our analysis. scMerge also requires users to input the cluster number in each single-cell sample. Even though scINSIGHT takes relatively more time than the other methods, its performance is still acceptable to server users. Its overall running time for datasets with 3,000 cells (6 samples), 4,680 cells (2 samples), 6,350 cells (48 samples), 9,741 cells (13 samples), and 71,638 cells (2 samples) are 4.5h, 0.9h, 2.8h, 2.9h, 36.4h, respectively. Given that scINSIGHT shows superior accuracy in identifying cellular identities in both simulation and real data studies, we think the additional computational time is worthwhile in return for increased accuracy and the unique condition-specific gene module information.*

*Table R1 Running time and memory usage of scINSIGHT and the other six integration methods. For scINSIGHT, the recorded time on the top was time used to run scINSIGHT with different values of K, and the recorded time on the bottom (in the parentheses) was time used to select parameters.*

*To address the reviewer’s question about scINSIGHT’s performance on larger data, we added a comparison based on a dataset of mouse retina (Dataset 7 from Tran et al.). This dataset includes two single-cell samples from mouse retina, with 12 cell types and 71,638 cells in total. The computation time and memory usage of scINSIGHT was 36.4h and 49.5G, respectively. As explained in our response to the reviewer’s comment 4, integration of these two single-cell samples is not a target application of scINSIGHT, since the two samples are from the same biological condition (healthy mouse retina). Nevertheless, scINSIGHT, Seurat, and Scanorama achieved similar adjusted Rand index, ranking second after Harmony. In addition, scINSIGHT was among the top three methods in terms of integration score (Figure R7).*

*In addition, we have two suggestions for users to further reduce the computational time of scINSIGHT given larger-scale data. First, users can assign single cells to major cell types with well-known marker genes, and then apply scINSIGHT to single-cell samples of the same major cell type. Second, users can apply the Metacell4 method separately to each single-cell sample to group statistically equivalent cells into metacells, thus reducing cell number in each sample, and then apply scINSIGHT to samples of metacells.*

*The above results and points are discussed on pages 15-16 of the revised manuscript.*

5. Simulation data are simple cases, need more variations. In simulation data, what happens when the shared cell types is minimal? For example, if only 1 out of 5 cell types are shared? Also, what happens if you change the proportions of the different cell types and make some more common and some cell types rare?

*Response: We thank the reviewer for the helpful suggestions on our simulation study. We agree with the reviewer that these variations will help better understand the performance of scINSIGHT in various scenarios. We have designed three variants of the original simulation data. Variant 1: only one cell type (C1) was shared by the single-cell samples; Variant 2: there existed a rare common cell type (the cell number of cell type C2 was reduced from 100 to 20 in each sample); Variant 3: there existed a rare condition-specific cell type (the cell number of cell type C4 was reduced from 100 to 20). For each simulated dataset, we compared scINSIGHT with the other six methods (Seurat, LIGER, Harmony, scMerge, scGen, and Scanorama). Our results show that scINSIGHT achieved the second highest adjusted Rand index after Scanorama in both variant 1 (Figure R8) and variant 3 (Figure R10). In variant 2 (Figure R9), scINSIGHT’s clustering accuracy was similar to that of Seurat and LIGER and slightly lower than Harmony and Scanorama, but the common gene modules identified by scINSIGHT led to high-quality lower-dimensional visualization (Figure R9B). It is also worth noting that, even though Scanorama had slightly better performance than scINSIGHT in the three variants of the simulation study, scINSIGHT outperformed Scanorama on all real datasets, especially on the melanoma and COVID-19 datasets. In summary, the above results demonstrate scINSIGHT’s ability to integrate heterogeneous single-cell samples and identify cellular identities in diverse settings. The new analysis and results are described on page 7 of the revised manuscript*

**Second round of review**

**Reviewer 2**

The reviewer appreciates that the authors made significant effort to address the questions raised in the initial submission, including adding more methods for benchmarking, and more real & simulated conditions for comparison. Below are some comments that hope to help:

1. When mentioning the unique features of scINSIGHT vs iNMF (used in LIGER), it says “iNMF” requires the same number of common and data-specific modules. However, the group of LIGER seems to have developed an updated version called UINMF that allowed unshared features: https://www.biorxiv.org/content/10.1101/2021.04.09.439160v1

2. I am intrigued by the poor performance of scGAPS, and the author mentioned that rather than integration, scGOGAPS does feature transferring (?), can you comment on how one might infer common features between 2 relative conditions? Which data set as source data and which one to be applied on, or the order does not matter?

3. Since there are multiple datasets and simulated conditions, it is probably good to make a heatmap that illustrate the result comparison of different metrics (for example, in rankings). This will give a clearer view. My impression is that scINSIGHT has variable rankings and is not the best method in some conditions.

4. I am intrigued by the broad variations of the results on scanorama, and scMerge in Figure R5 compared to other plots. Can you explain?

5. For Dataset 7 of Tran et al, they do not appear to be just batches (the original paper called it “batch”). They are the same tissues from different animals by different labs, therefore this reviewer thinks they can be considered “relevant but not same conditions”.

6. The running time and memory use of scINGIGHT seem to be significantly more than other methods (1 order or more). It is also intriguing that LIGER takes so long to compute when the original authors claimed that iNMF is very efficient.

7. The results in the new simulations do not support the conclusion that scINSIGHT is the best method.

**Authors Response**

**Point-by-point responses to the reviewers’ comments:**

Editors: Basically, we would ask you to modify the manuscript to include more details about the novelty of the software and the experiments, as you’ve written them in the rebuttal letter. We’d ask that you emphasize the conditions with scINSIGHT performs better, even if not on all conditions.

Response: We thank the editors for the above suggestions. We have revised the manuscript to better explain the novelty of scINSIGHT and its improvements over existing methods. The revisions are highlighted in red in the revised manuscript (pages 2, 3, and 18-20). The novel features and advantages of scINSIGHT are now emphasized in several sections, including Introduction (pages 2-3), first section of Results (page 3), and Methods (pages 18-20). According the suggestions of the editors and reviewer 2, we have made a new figure to compare the overall rankings and relative performance of scINSIGHT and the other methods in terms of clustering accuracy and integration score (please see Figure R1 below). In each heatmap, the methods are ranked by the average relative performance across all datasets. We can see that scINSIGHT is the best performing method in both heatmaps. These comparisons demonstrate the strengths of scINSIGHT to integrate and analyze single-cell samples from different biological conditions. Specifically speaking, in terms of clustering accuracy, scINSIGHT and Scanorama had the highest and almost the same clustering accuracy in simulations, while scINSIGHT had much better accuracy than Scanorama in real data applications; in terms of integration score, scINSIGHT has the best average score, followed by Harmony and scMerge. These new results have been added to pages 16-17 of the revised manuscript.

Reviewer #1:

*Response: We thank the reviewer for acknowledging the importance of our work. We have revised the manuscript based on the reviewers’ comments, and the revised contents are marked in red in the updated manuscript. Please see our point-by-point response below.*

Minor comments: 1. Maybe I missed it, but I still do not see how these common and condition-specific pathways are being defined.

*Response: In our last revision, we have changed the term from “gene pathway” to “gene module” to avoid confusions. An explanation of this concept is given on both page 3 and page 18. In this revision, we have updated the descriptions in Methods to clarify the definition of gene modules with mathematical notations. Please see page 18 of the revised manuscript.*

2. Code works now - although with each iteration it throws a warning. Also, code only works if you load their pre-calculated scINSIGHT object - the visualization code breaks if you run the scINSIGHT algorithm yourself.

*Response: We thank the reviewer’s feedback on the software package. We have fixed the warnings and errors, and tested the package on both Linux and Mac OS systems. We will continue to maintain the package and users can submit any questions or issues through the Github page of scINSIGHT*

3. Co-expression experiments - The absolute correlation coefficient values are low, which is not too surprising. Differences between within- and across-group correlation sets appear more extreme in condition-specific modules relative to common modules, which is probably expected.

*Response: We thank the reviewer for taking the time to review this newly added analysis. We agree with the reviewer. Genes belonging to the same module are expected to have similar biological functions, so their gene expression profiles are more similar than genes belonging to different gene modules. However, since they are different genes and singlecell gene expression data are sparse, it is expected that the absolute correlation values are not very large.*

4. Empirical modeling point was ignored or not understood.

*Response: As we have explained in our last revision, the gene sampling and swapping approach used in PMID=29491377 does not seem to be applicable in our studies. Therefore, we designed three variants of the original simulation study to further evaluate the accuracy and robustness of scINSIGHT in different scenarios. scINSIGHT is among the best two methods in these simulation studies. These analyses are described on page 7 of the revised manuscript.*

Reviewer #2: The reviewer appreciates that the authors made significant effort to address the questions raised in the initial submission, including adding more methods for benchmarking, and more real & simulated conditions for comparison. Below are some comments that hope to help:

*Response: We thank the reviewer for taking the time to review our revision. We have revised the manuscript based on the reviewers’ suggestions, and the revised contents are marked in red in the updated manuscript. Please see our point-by-point response below.*

1. When mentioning the unique features of scINSIGHT vs iNMF (used in LIGER), it says “iNMF” requires the same number of common and data-specific modules. However, the group of LIGER seems to have developed an updated version called UINMF that allowed unshared features.

*Response: We thank the reviewer for bringing this new method to our attention. In our manuscript, we have highlighted three methodological limitations of iNMF that are addressed by the proposed scINSIGHT method (pages 19-20). In the above comment, the reviewer mentioned a method named UINMF and suggests that it is able to address one of these limitations.*

*After carefully reading the UINMF preprint, we found that the UINMF method focuses on a completely different question and does not address any of the above three limitations of iNMF (used in LIGER). As an extension of the LIGER method, UINMF was proposed to integrate single-cell multi-omic datasets when datasets of multiple modalities have different features. For example, the scRNA-seq data only have gene features while the snATAC-seq data have both gene and intergenic features. However, in the problems of integrating multiple scRNA-seq datasets, since all the datasets only have gene features, UINMF will reduce to the LIGER method. In our previous manuscript, we have already compared the performance of scINSIGHT, LIGER, and five other integration methods, which shows that scINSIGHT has the best overall performance in terms of both clustering and integration (please also see our response to the reviewer’s comment 3).*

2. I am intrigued by the poor performance of scGAPS, and the author mentioned that rather than integration, scCOGAPS does feature transferring (?), can you comment on how one might infer common features between 2 relative conditions? Which data set as source data and which one to be applied on, or the order does not matter?

*Response: We are sorry about the confusion. We didn’t mention or suggest that “scCOGAPS does feature transferring”. The main reason why scCoGAPS had poor performance is that it was not designed to directly take multiple scRNA-seq datasets as its input. In the scCoGAPS publication, the authors used another method named ProjectR to project new datasets to the latent space identified by scCoGAPS (using a source dataset). Since this approach requires the selection of a source dataset, which is not straightforward in applications studied in our work, we had to combine the datasets into a single read count matrix before applying scCoGAPS. Given this key difference between scCoGAPS and the other methods, we have decided to not include its results in the manuscript to avoid confusion.*

3. Since there are multiple datasets and simulated conditions, it is probably good to make a heatmap that illustrate the result comparison of different metrics (for example, in rankings). This will give a clearer view. My impression is that scINSIGHT has variable rankings and is not the best method in some conditions. 7. The results in the new simulations do not support the conclusion that scINSIGHT is the best method.

*Response: We thank the reviewer’s suggestion to more clearly illustrate the comparison of different methods. We have made a new figure to compare the rankings and relative performance of scINSIGHT and the other methods in terms of clustering accuracy and integration score (Figure R2). In each heatmap, the methods are ranked by the average relative performance across all datasets. We can see that scINSIGHT is the best performing method in both comparisons.*

*In comment 7, the reviewer mentioned the comparison based on the simulated datasets. From Figure R2A, it is clear that scINSIGHT and Scanorama had almost the same clustering accuracy in the four simulations, even though scINSIGHT did not have the highest score on every dataset. In real data applications, scINSIGHT had much better accuracy than Scanorama.*

*The above new results have been added to pages 16-17 of the revised manuscript.*

4. I am intrigued by the broad variations of the results on scanorama, and scMerge in Figure R5 compared to other plots. Can you explain?

*Response: We thank the reviewer for questions about these two methods. For scMerge, we found that whether or not to allow scMerge select highly variable genes will significantly impact its results. Therefore, we have updated the analyses to let scMerge internally select gene features. After this revision, scMerge had more consistent performance across different datasets (Figure R2). On the COVID-19 dataset, scMerge performed similar to Seurat and scGen (Figure R3). On the wound healing dataset, scMerge had slightly lower median Silhouette scores than Seurat and Harmony (Figure R4). On the melanoma dataset, the scMerge package encountered errors, so its results were not obtained. The above revision has been added to the updated manuscript (Figures 5 and 7).*

*For Scanorama, to investigate why it had poorer performance on the COVID-19 dataset, we performed principal component analysis (PCA) on its integrated data. The results of PCA suggested that Scanorama was not able to distinguish between the sample-specific variation and the cell-type-specific variation in this dataset, so the integrated data were over-smoothed (Figure R5).*

5. For Dataset 7 of Tran et al, they do not appear to be just batches (the original paper called it “batch”). They are the same tissues from different animals by different labs, therefore this reviewer thinks they can be considered “relevant but not same conditions”.

*Response: We thank the reviewer for this comment. We have revised the first paragraph of Methods to clarify the assignment of biological conditions. The revised description has been pasted below.*

*“We propose a matrix factorization model named scINSIGHT to jointly analyze multiple single-cell gene expression samples belonging to different biological conditions (i.e., groups). The comparison between these conditions should be of biological interest, and these biological conditions can be assigned based on phenotypes or experimental conditions, such as different developmental stages, disease phases, or treatment groups.”*

*As the reviewer has pointed out, the two single-cell samples in Dataset 7 of Tran et al were from the same tissue type but different animals. Since both samples came from the same healthy tissue type, it is more proper to treat them as two samples from the same biological group, instead of samples from different groups. This also explains why the original paper called them “different batches”.*

6. The running time and memory use of scINGIGHT seem to be significantly more than other methods (1 order or more). It is also intriguing that LIGER takes so long to compute when the original authors claimed that iNMF is very efficient.

*Response: We compared all methods using the same computing environment, so the comparison was fair to all methods. The reviewer has questioned about LIGER’s efficiency. In our comparison, LIGER finished running within 700s except for the dataset with 71,638 cells, which took it 14,911s. This suggests that LIGER is indeed efficient when datasets are not very large.*

*In the manuscript, we also pointed out that scINSIGHT’s memory usage is on the medium level among the seven methods, and scINSIGHT is admittedly slower than the other methods because it uses a more complex model to obtain condition-specific information not available from the other methods. Even though scINSIGHT takes relatively more time than the other methods, its performance is still acceptable to server users. Its overall running time for datasets with 3,000 cells (6 samples), 4,680 cells (2 samples), 6,350 cells (48 samples), 9,741 cells (13 samples), and 71,638 cells (2 samples) are 4.5h, 0.9h, 2.8h, 2.9h, 36.4h, respectively. Given that scINSIGHT shows superior accuracy in identifying cellular identities in both simulation and real data studies, we think the additional computational time is worthwhile in return for increased accuracy and the unique condition-specific gene module information.*
